# Adapting the Voicing My CHOiCES Advance Care Planning Communication Guide for Australian Adolescents and Young Adults with Cancer: Appropriateness, Acceptability, and Considerations for Clinical Practice

**DOI:** 10.3390/cancers15072129

**Published:** 2023-04-03

**Authors:** Ursula M. Sansom-Daly, Megan Zhang, Holly E. Evans, Jordana McLoone, Lori Wiener, Richard J. Cohn, Antoinette Anazodo, Pandora Patterson, Claire E. Wakefield

**Affiliations:** 1 School of Clinical Medicine, UNSW Medicine & Health, Randwick Clinical Campus, Discipline of Paediatrics, UNSW Sydney, Kensington, NSW 2052, Australia; 2 Behavioural Sciences Unit, Kids Cancer Centre, Sydney Children’s Hospital, Randwick, NSW 2031, Australia; 3 Sydney Youth Cancer Service, Nelune Comprehensive Cancer Centre, Prince of Wales Hospital, Randwick, NSW 2031, Australia; 4 National Institutes of Health, Bethesda, MD 20892, USA; 5 Kids Cancer Centre, Sydney Children’s Hospital, Randwick, NSW 2031, Australia; 6 Canteen Australia, Sydney, NSW 2042, Australia; 7 Faculty of Medicine and Health, The University of Sydney, Sydney, NSW 2006, Australia

**Keywords:** advance care planning, adolescents and young adults, cancer, end of life, palliative care, communication

## Abstract

**Simple Summary:**

Adolescents and young adults (AYAs) with life-threatening illnesses want to voice their end-of-life choices. However, these conversations do not happen often. This is in part because of the discomfort that surrounds talking about these issues and because health professionals often have not had enough training in this area. *Voicing My CHOiCES* is an American booklet which serves as a communication guide to help AYAs have these important discussions with their families and health professionals and document their preferences for care. Our study looked at whether the American guide was suited to young Australians, and what aspects of the guide young people, health professionals, and parents thought caused stress. Overall, participants thought the guide was appropriate and helpful for adolescents and young adults, and they talked about different sources of stress for AYAs attempting to complete it. Our research will inform the adapted Australian *Voicing My CHOiCES* and support health professionals in how to use this guide to facilitate positive end-of-life outcomes for young people and their families.

**Abstract:**

**Background:** Adolescents and young adults (AYAs) with life-threatening illnesses need support to discuss and voice their end-of-life choices. Voicing My CHOiCES (VMC) is a research-informed American advanced care planning guide designed to help facilitate these difficult discussions. This multi-perspective study aimed to evaluate its appropriateness, acceptability, and clinical considerations for Australian AYAs with cancer. **Procedure:** Forty-three participants including AYAs who were either undergoing or recently completed cancer treatment, their parents, and multidisciplinary health professionals assessed the acceptability of each VMC section quantitatively (appropriateness—yes/no, helpfulness and whether content caused stress—1 = not at all, to 5 = very) and qualitatively (sources of stress). AYAs also assessed the benefit and burden of completing several sections of the document, to inform clinical considerations. We conducted a mixed-methods analysis to obtain descriptive statistics and to identify prominent themes. **Results:** In terms of acceptability, almost all participants (96%) rated VMC as appropriate overall. Perceived helpfulness to their situation (to themselves/their child/their patients), to others, and stressfulness were rated, on average, as 4.1, 4.0, and 2.7/5, respectively. Stress was attributed to individual and personal factors, as well as interpersonal worries. All sections were considered more beneficial than burdensome, except for the Spiritual Thoughts section (Section 6). **Conclusions:** While VMC is an acceptable advance care planning guide for AYAs with cancer, changes to the guide were suggested for the Australian context. Health professionals implementing VMC will need to address and mitigate anticipated sources of stress identified here. Future research evaluating the impact of a new culturally adapted Australian VMC guide is an important next step. Finally, the clinical implications of the present study are suggested.

## 1. Introduction

Adolescents and young adults (AYAs) with cancer—a group encompassing varyingly defined age ranges worldwide [1]—are challenged with juggling the emotional and physical burdens of their illness at a critical stage of development. The far-reaching psychosocial impacts this group experiences include delayed attainment of independence, interrupted formation of social relationships [2,3], and increased mental health disorders [4]. These AYAs often also need intensive and complex medical treatment with significant side effects. These combined challenges mean it is important that when end-of-life communication is needed, it is addressed in an age-appropriate and timely manner. Young cancer patients consider end-of-life concepts early in their illness, have important opinions regarding care throughout the cancer trajectory, and desire a place to voice these preferences [5,6,7,8]. Indeed, the early introduction of palliative care and end-of-life concepts has been suggested as a standard of care [9,10]. High-quality communication about sensitive end-of-life topics is therefore vital to help enhance the quality of life for AYAs whose survival is not guaranteed.

Advanced care planning is a process which aims to improve the quality of patients’ end-of-life period by supporting them to make choices consistent with their values and goals of care [11,12]. It empowers families to better understand and honour the individual’s preferences [13]. Advance care planning is generally considered worthwhile, acceptable, and feasible, and when carried out properly, does not increase anxiety, distress, or depressive symptoms among seriously ill adolescents [13,14,15]. Advanced care planning also enables individuals to maximise their quality of life and make the most of their remaining time [16]. It can be incorporated regardless of survival likelihood, level of suffering, or disease status [17]. Insufficient planning for AYA patients has been linked to poorer outcomes for the AYA and their family, including inadequate pain management, stress, and family decisional regret [13,18]. Research has also associated poor-quality, inadequate, and delayed communication with patients receiving more invasive procedures, prolonged use of active cancer treatments, more intensive care unit hospitalisations close to death [19], and deaths that occur in hospitals against the patients’ wishes [8].

AYAs generally want to be involved in end-of-life communication and have preferences for how this happens [20,21]. However, AYAs vary in their level of engagement with end-of-life conversations, with some AYAs (both healthy and those with life-threatening illnesses) not wishing to be involved in end-of-life decision making [22], or conversations [23]. Barriers for AYAs include concerns that these conversations may be perceived as representing a loss of hope, may precipitate treatment discontinuation [24,25], or may negatively impact relationships with their health professionals [26]. AYAs may also worry about inducing family distress [24], while families may avoid mentioning advance care planning to maintain a positive or supportive stance [25]. This can lead to a ‘conspiracy of silence’ where neither desire to initiate these conversations [24]. As such, advance care planning and end-of-life communication in AYA patients often occurs infrequently, with research suggesting that it occurs among as few as 3% of AYAs without clinician guidance [14], and too late to facilitate meaningful choices regarding decision making, palliative care, and psychosocial support [27,28,29,30,31]. Clinician initiations of end-of-life discussions are, therefore, often preferred [26]; however, clinical guides and training to support health professionals to broach these difficult discussions are limited.

Despite these well-recognised barriers and evidence of high AYA and family engagement in advance care planning when evidence-based guides or resources are used [14], there are few age-appropriate guides available to help AYAs to communicate their end-of-life preferences. *Voicing My CHOiCES* (VMC), a research-informed AYA advance care planning guide developed in the United States (US) [25], aims to provide communicative support for AYAs and has been found to be easy-to-understand, age-appropriate, helpful, anxiety-reducing and worthwhile by AYAs and health professionals [7,27,32,33,34,35]. Furthermore, VMC may also be an effective conversational structure to support health professionals’ skill development: just one simulated session with VMC significantly increased nurse practitioners’ confidence in facilitating AYAs’ advance care discussions [33].

Cultural differences frequently impact advanced care planning for young people [36] and can decrease the sensitivity of end-of-life communication [7]. Australia and the US share similar Westernised healthcare systems that employ multidisciplinary approaches to AYA care [37,38], with a family-focused model of care that transitions with age to prioritise patient autonomy. However, even countries with similar healthcare cultures and languages may have subtle but important cultural and linguistic differences [7,39] that can affect end-of-life communication quality and decrease the acceptability of advanced care planning guides like VMC.

Culturally-tailored guides are essential for patient-centred care; more comprehensive research on VMC’s acceptability and appropriateness among various populations of AYA cancer patients will aid the development of culturally-appropriate advanced care planning guides for multiple global populations. In developing communication resources appropriate to different local contexts, it is important to learn from international efforts to tailor advanced care planning interventions. For example, Walczak and colleagues adopted an adult advance care planning intervention amongst Australian and US cancer cohorts and found several differences in prognostic and end-of-life communication, including increased euphemism use in the US cohort compared to the Australian group [39].

We have previously reported early pilot data highlighting that Australian AYAs perceived VMC may need linguistic adaptation to better reflect the more secular Australian culture and to address the different cultural and religious diversity and composition of the Australian population [7]. Here we describe in detail the first stage of tailoring the VMC advance care planning guide for AYAs to suit the Australian clinical context, by examining its acceptability and perceived sources of stress from multiple stakeholder perspectives, to inform its adaptation and clinical use in the Australian context. Our results will inform later study phases focused on adapting and implementing a culturally-tailored VMC with Australian AYAs. Using a multi-perspective sample to examine the views of AYAs, their family members, and treating health professionals, we aimed to answer the following research questions:

1. How acceptable is the American VMC guide for Australian AYAs?
2. What aspects of the VMC guide are considered stressful, beneficial, and burdensome?
3. How appropriate is the VMC guide for the Australian clinical context?

## 2. Methods

### 2.1. Inclusion and Exclusion Criteria

Three participant groups with important perspectives on end-of-life conversations with AYAs were eligible: AYAs with a cancer history (both patients currently undergoing active cancer treatment as well as survivors who had completed treatment), parents (both of of AYA cancer patients/survivors, and bereaved parents), and multidisciplinary health professionals caring for AYAs with cancer (who had treated ≥1 AYA with cancer who had died). The Australian AYA age range (15–25-year-olds [40]) was used to ensure the ecological validity of our results within the Australian clinical context. Participants who were (1) too medically unwell, cognitively impaired, or within six months of diagnosis, (2) unable to read English, or (3) unable to provide informed consent, were excluded.

### 2.2. Recruitment

A purposive sample [41] of AYAs, parents, and health professional stakeholders was recruited. AYAs were recruited from one adult and one paediatric hospital in Sydney, Australia, via local health professionals. Parents were recruited via the above methods, as well as via local community AYA cancer support services and a bereavement study database. Health professionals were recruited via the national network of AYA-specific Youth Cancer Services [42], Australia, and were unrelated to the AYA and parent samples.

### 2.3. Research Design

This study used a cross-sectional, single timepoint design using semi-structured interviews, which were subjected to mixed-methods (quantitative and qualitative) data analysis. This mixed-methods multi-perspective approach aimed to facilitate a comprehensive assessment [43] of the complex views that AYA patients and other stakeholders may have towards considering VMC and sensitive end-of-life topics.

### 2.4. Procedures

All participants gave their informed consent for inclusion before they participated in the study. The study protocol was approved by the Ethics Committee of the South-Eastern Sydney Local Health District (Reference number 15/198). Following opt-in, we obtained demographic information via a paper questionnaire administered in person prior to the start of the interview. Data was then collected through semi-structured face-to-face interviews that explored helpfulness, perceptions of stress, and sources of stress, as well as the benefits and burdens of completing several VMC pages. The interviews were administered by psychology-trained research staff. We adapted Wiener and colleagues’ interview protocol [27], to standardise our approach to data collection and facilitate later cross-country data comparisons. Participants who were unable to travel completed the demographics questionnaire online via Qualtrics and completed the interview via telephone.

After a brief introduction to VMC overall and to each section (Table 1), participants were asked to assess each section’s acceptability by evaluating the appropriateness, helpfulness to themselves, helpfulness to hypothetical others in their situation, and stress caused by the guide. Appropriateness measures were designed with a binary “yes”/”no” response; however, as several participants’ response was “unsure”, this was added as a category during analysis. Helpfulness and stressfulness were measured on a 5-point Likert scale (e.g., 1 = not at all stressful, to 5 = very stressful). To explore the factors contributing to perceived stress, participants who rated stress 2 or above were asked “*What would be stressful about thinking about these questions*?”. Participants also repeated helpfulness and stressfulness assessments for two proposed hypothetical VMC additions suggested by Wiener et al. [44,45]: Section 9—Online Accounts and Section 10—VMC Storage (see Table 1).

Participants were also asked to comment on aspects of the guide they would add, remove, or otherwise change. AYAs further completed three VMC sections including at least Section 3—Medical Decisions followed by two sections of their choice. They reported the benefit and burden of completing each from 0 to 4 (0 = not at all beneficial to me, 4 = very much beneficial to me), and provided a reason for their rating via a multiple-choice question. For example, participants could select that completing the page was burdensome because “the questions were confusing”. Interviews were audio-recorded and transcribed verbatim.

### 2.5. Data Analysis

We calculated descriptive statistics including means, standard deviations, and ranges using IBM SPSS Statistics 26. We analysed qualitative data via thematic analysis [46], which involved MZ and HEE reading all transcripts. Following this, MZ identified key themes and formed preliminary coding trees for each section. These themes were then discussed among co-authors, refined, and grouped under higher-level concepts to form one common coding tree. We then coded each transcript using NVivo 12.6.0. Codes, themes, and concepts were rediscussed, reviewed, and refined by USD, MZ, HE, and JM, and the final codes were used as a framework to present the qualitative data.

## 3. Results

### 3.1. Participant Demographics

Forty-three participants, comprising 10 AYA cancer survivors, 5 parents of AYA cancer patients, and 28 health professionals were interviewed between December 2015 and November 2019 (Table 2). Our AYA/parent sample was predominantly female, with AYAs reporting a range of cancer diagnoses and prognoses (Table 2). The three most prevalent diagnoses in our sample by frequency were Acute Myeloid Leukaemia (*n* = 3), Osteosarcoma (*n* = 2), and Hodgkin’s Lymphoma (*n* = 2).

Our health professional sample represented a mix of disciplines. Health professionals also reported having considerable experience with end-of-life care in AYA cancer, with almost two-thirds having cared for more than 15 AYAs who had died from their cancer (see Table 3).

### 3.2. Research Question 1: How Acceptable Is the American VMC for Australian AYAs?

#### 3.2.1. Acceptability: Appropriateness of Content

Overall, 96.2% of participants considered VMC to be appropriate. Sections on Comfort, Family and Friends, and My Voice were rated as appropriate by almost 100% of participants. Life Support was rated as least appropriate overall but was still rated as appropriate by 70% of participants.

**Figure 1:**
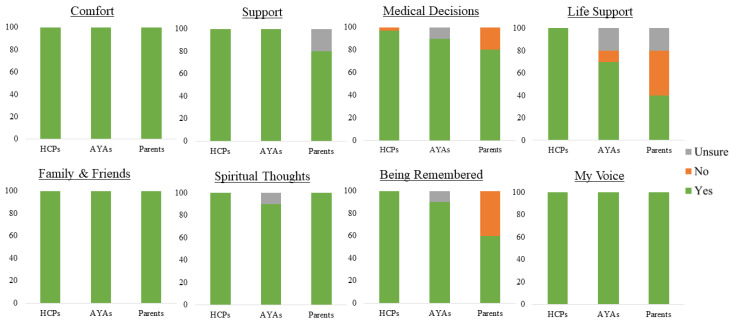
Appropriateness of Voicing My Choices content by section, according to different participant groups. **Abbreviations**: **HCP**—Healthcare professional, **AYA**—Adolescents and young adult, **VMC**—Voicing My CHOiCES^TM^.

In terms of different participant groups, VMC’s appropriateness was rated highest by health professionals (99.6%), who almost unanimously rated all sections as appropriate, with only one “not appropriate” response for Medical Decisions (Figure 1). This was followed by AYAs (92.5%), who rated all sections as appropriate, except for Life Support (70%). Parents reported the lowest ratings of overall appropriateness (82.5%), with Life Support and Being Remembered being rated as appropriate by only 40% and 60% of parents, respectively.

Figure 1 depicts perceived appropriateness of VMC content by section. Appropriateness was not analysed for sections on VMC storage and the AYAs’ online presence as these were hypothetical new proposed VMC additions. Percentages were calculated after excluding missing participant responses from the cohort (data missing for *n* = 1 HCP and *n* = 1 parent in My Voice, and *n* = 1 parent in the Life Support section) 

#### 3.2.2. Acceptability: Helpfulness 

**Figure 2:**
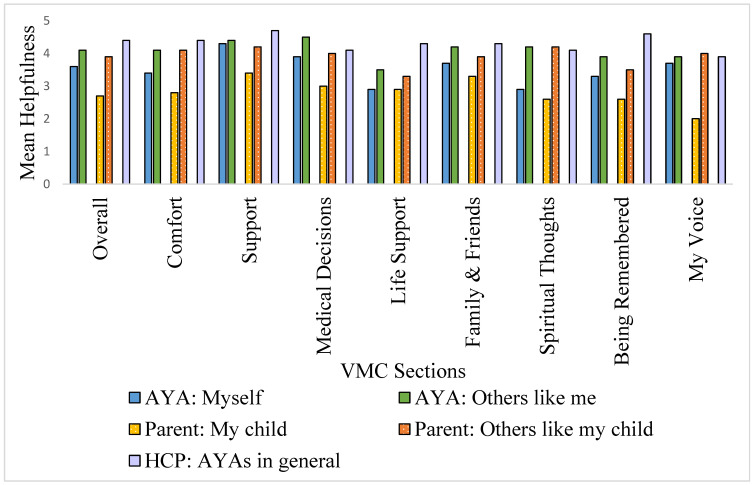
Helpfulness of VMC sections to oneself versus others like me (AYAs), and my child versus others like my child (parents), and AYAs in general (health professionals).

VMC’s helpfulness was rated, on average, as helpful to very helpful (mean of 4.1/5 overall), when considering themselves (AYAs), their child (parents), or AYA patients in general (health professionals). Across all groups, support was considered the most helpful (M = 4.5), while Spiritual Thoughts and My Voice were the least helpful (means 3.6, 3.7, respectively; Figure 2). Averaging across all sections, helpfulness was rated highest by health professionals (with a mean rating falling in the *helpful to very helpful* range, M = 4.4/5), followed by AYAs (*somewhat helpful to very helpful*; M = 3.6) and parents (*little to somewhat helpful*; M = 2.7).

We also examined helpfulness data according to each individual VMC section. For AYAs and parents, the Support and Medical Decisions sections were rated the most helpful (both receiving means of 4.3/5, in the *helpful to very helpful* range), while Life Support and Being Remembered were considered the least helpful by comparison, though both still received mean scores in the *somewhat helpful to very helpful* range (Life Support mean = 3.4/5, Remembrance, mean = 3.7/5). While AYAs and parents thought VMC would be helpful for themselves or their child (*somewhat helpful to helpful*; means 3.6/5 and 2.7/5, respectively), both groups rated helpfulness for others significantly higher (*helpful to very helpful*; means 4.1/5 and 3.9/5, respectively). My Voice was the only section that AYAs rated less helpful than parents.

#### 3.2.3. Acceptability: Perceived Stress

**Figure 3:**
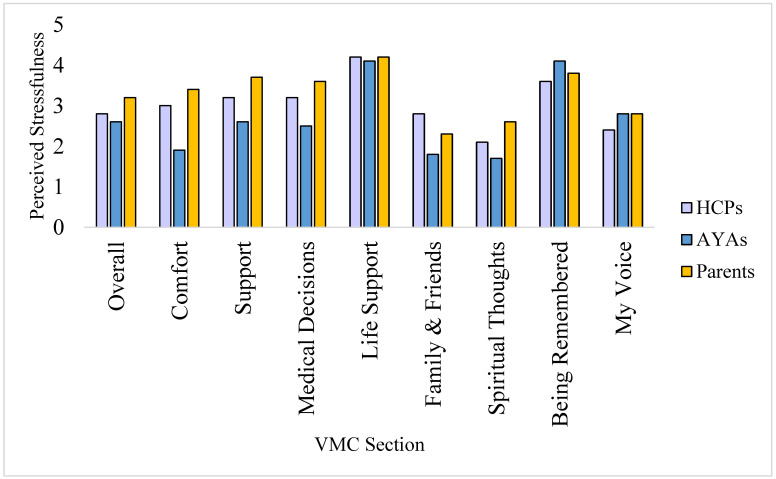
Stress of Voicing My Choices sections by participant group. **Abbreviations**: **AYA**—Adolescents and young adults, **HCP**—Healthcare professionals, **VMC**—Voicing My CHOiCES. Participants assessed stressfulness from 1 to 5 (1 = not at all stressful, 2 = a little stressful, 3 = somewhat stressful, 4 = stressful, 5 = very stressful).

Overall, VMC was perceived as *a little to somewhat stressful*, rated with a mean of 2.7/5. Parents anticipated the greatest degree of stress in completing VMC (mean of 3.2/5, *somewhat stressful*), followed by health professionals and AYAs themselves (*a little stressful to somewhat stressful*; mean = 2.8 and 2.6/5, respectively). However, parents and health professionals demonstrated wider variability in their ratings of perceived stress ratings across the 10 sections compared with AYAs. Examining which VMC content was associated with the greatest perceived stress, Life Support and Being Remembered were rated the most stressful (Life support, *stressful to very stressful*, M = 4.2, and Being Remembered, *somewhat stressful to stressful*, M = 3.7/5), with Being Remembered being the only section which AYAs rated more stressful than both parents and health professionals (Figure 3). Stress was lowest for the sections on VMC Storage and Spiritual Thoughts (means 1.7 and 2.0/5, respectively).

### 3.3. Research Question 2: What Aspects of the VMC Guide Are Considered Stressful, Beneficial, and Burdensome?

#### 3.3.1. Anticipated Sources of Stress

We coded the sources of perceived or anticipated stress described by participants into four categories: (i) individual patient factors, (ii) AYA-related developmental factors, (iii) stress regarding the anticipated impact on family and friends, and (iv) stress from interactions with others about the VMC guide. Table 4 shows selected illustrative quotes, while Supplementary Table S1 presents all quotes underpinning each theme in full.

(i) Individual factors

Health professionals highlighted how individual factors such as AYAs’ communication and coping style, disease-related knowledge, and prognostic awareness could influence how AYAs approached and processed the topics raised by VMC and how stressful it may be to use the guide:

“I wouldn’t be doing a page like this with someone who did not know their treatment was not curative, … someone who isn’t comfortable to talk about it.”

(Female health professional (HCP), 39 years)

AYAs and parents similarly raised the idea that some AYAs may simply not feel ready to discuss these topics, or may not feel that having these conversations would help them cope with their situation:

“When I was going through treatment, it never once crossed my mind—about a funeral or what’s going to happen to me once I pass away. … If you’re going through treatment [those thoughts] can be quite overwhelming and just quite negative. … I’d rather focus on [the] positive. It sort of doesn’t give you any hope when you’re confronted with questions like that.”

(Female AYA, 24 years)

(ii) AYA-related developmental factors

Participants reflected on how AYAs’ cognitive, social, and emotional development may contribute to stress around end-of-life decision making. They showed that the confronting nature of the topics contained in VMC encountered perhaps for the first time in their lives, could be stressful to consider.

“Then at that age when that’s all you can do, when you’re being asked ‘so when you want to die, where do you want to die.’ It’s like—‘but hang on a minute, I want to go to the movies with my friends’.”

(Female bereaved parent, 52 years)

“Probably the last question ‘when the end of my life is near’, … I think no matter how you frame that it’s going to create some stress for the young person.”

(Female HCP, 37 years)

Some suggested that the demands of engaging with adult decision making may be challenging for some AYAs and feel that this contrasts with the unequal doctor-patient power dynamic they may have grown up with:

“You’re brought up… you go to the doctor, and you do what the doctor tells you. So, to be able to stop previously started treatment, or to ‘hire or fire a healthcare worker’—that’s stuff that’s like, wow—hold on—that’s a bit… [intimidating]”

(Female parent, 52 years, referring to the Medical Decisions page)

(iii) Concerns around the potential impact on family and friends

Participants suggested that AYAs’ tendency to consider others in their actions (or not) may be a source of stress for AYAs completing VMC—for example, by worrying about the impact of their responses on family and friends:

“It also has the scope to make you feel concerned about how your loved ones are going to be feeling after your death, and that can be really upsetting.”

(Male HCP, 49 years)

(iv) Interpersonal interactions around the anticipated stress of HCPs using the guide

Participants also thought that broader social issues also influenced the anticipation or stress of completing VMC. Participants noted that stress could arise due to the social complexities within the AYA’s interpersonal network, for example, if the AYA-nominated visitors, medical decision makers, and those to whom online-account access would be granted were in conflict with the family’s preferences.

“I’m sure there’s going to be young people who don’t want their default, their parent or guardian, to be making those decisions.”

(Female HCP, 27 years)

Participants also noted that stress could arise where AYAs and/or their family members were not ready to have these conversations together:

“Sometimes because the parents or the siblings can’t get there [to accepting the possibility of death] the child feels… [that] they don’t have permission to get there, and I think that permission to die … and permission to express your feelings before you die is very important.”

(Female HCP, 32 years)

As one health professional noted, successful engagement with the guide and a reduction in such distress would be “… dependent on you setting the scene and having that person walking you through it, someone else who can give it [VMC] with [an] explanation rather than just throw it [to them] as a questionnaire.”

(Female HCP, 49 years)

#### 3.3.2. Experience of Using the VMC Guide: Benefit and Burden

All AYAs (*n* = 9) completed Medical Decisions and rated it as moderately beneficial (*M* = 2.89/4, *SD* = 1.27, range = 0–4) and only a little burdensome (*M* = 1.13/4, *SD* = 1.13, range = 0–3; Supplementary Table S2). The two most-frequently endorsed benefits identified for this section were “Helped me think about/decide what I want” (*n* = 6), and “It was helpful/relieving to voice my thoughts” (*n* = 5), while the two most-endorsed reasons for the burden were “Thinking about the topics discussed made me anxious” (*n* = 3) and “Questions were confusing” (*n* = 2).

AYAs subsequently chose a range of other VMC sections to complete, with the Comfort, Life Support, and Being Remembered sections chosen equally frequently (by *n* = 4). AYAs found all sections more beneficial than burdensome (Supplementary Table S3). Across all pages completed, the mean benefit rating was 3.1 (*SD* = 0.29), and the mean burden rating was 0.9 (*SD* = 0.12). The top three benefits named were “It was helpful/relieving to voice my thoughts” (*n* = 15), “Helped me think about/decide what I want” (*n* = 14), and “It provided me the opportunity to discuss my wishes with others” (*n* = 12). The top three sources of burden were “Thinking about the topics discussed made me anxious” (*n* = 9), “The questions were difficult to answer” (*n* = 4), and “The questions were confusing” (*n* = 4). All sections (aside from the My Voice section) were chosen to be completed by at least one AYA.

### 3.4. Research Question 3: How Appropriate Is the VMC Guide for the Australian Context?

Overall, there were 159 suggested changes to the US VMC, with an average of 17 changes per page. All participants suggested at least one change. My Voice has the least suggested changes at 10, and Life Support had the most at 27.

Changes included those surrounding differences in terminology between Australian and US health systems, e.g., the use of the terms “Health care agent”, “DNR”, “Medicaid”, and reference to the option of “hiring and firing” healthcare professionals. One health professional commented on the American language on the Medical Decisions page, “*I’m going to say somewhat helpful because there’s an American connotation to a lot of the words so young people from Australia probably wouldn’t understand some of the wording.”* (Female HCP, 34 years).

Participants also noted the potential for a hypothetical digital, internet-mediated, or app-based version of the guide in the future, as opposed to the current paper-based booklet format. In this context, participants suggested changes including updates for modern technology, the addition of suggestions for photos and videos as part of How I Wish to Be Remembered, and suggesting writing the My Voice content electronically, or as a voice recording on a mobile phone. One healthcare professional suggested the inclusion of podcasts as an option for How I Want to Be Comforted, *“In my experience with young people, and as technology goes on and things like that, you know, it might be just not as appropriate to use book stories or readings. Readings has a bit of a religious connotation, I guess, to it, so whether that could be changed to maybe the kind of movies, something that’s more technologically related or my favourite podcast.”* (Female HCP, 34 years).

Participants also commented that aspects of the graphic design of the guide were not especially representative of Australia, for example, the water, reeds, and dragonfly motif.

“*I think again dragonflies are really—I haven’t been to the (United) States a lot. There are a lot dragonflies around. More than you see here, I don’t know why, and so maybe it’s more—it has a connotation for them but I don’t know about it.*”

(Female HCP, 46 years)

Some participants also expressed that they liked the blue and green colour scheme, while many disliked the mix and arrangement of fonts.

## 4. Discussion

Age-appropriate advance care planning communication guides can facilitate best-practice end-of-life communication with AYAs with cancer. This multi-perspective study evaluated the acceptability of the VMC guide for Australian AYAs with cancer to inform the guide’s cultural and linguistic adaptation to local use. This was the first study to evaluate VMC’s acceptability and feasibility in Australia, with diverse health professionals embedded within paediatric and adult hospitals, and the first to include parent feedback. We found that AYAs, parents, and health professionals for the most part considered VMC appropriate and helpful for Australian AYAs with cancer. Our results accord with previous US-based evaluations amongst AYAs with cystic fibrosis and their health professionals [34], bone marrow transplant health professionals [32], nurses [33], and AYAs with cancer [27], who have rated VMC as easy-to-understand, age-appropriate, disease-appropriate, helpful, and worthwhile.

Some nuances around VMC’s perceived helpfulness emerged from our data. Health professionals rated VMC’s appropriateness and helpfulness higher than AYAs and parents, while both AYAs and parents considered VMC more helpful to (hypothetical) others than to themselves. This tendency to consider VMC more helpful in others has been shown before. For example, 85% of health professionals working in cystic fibrosis found VMC useful for their patients, compared to 100% for “someone with cystic fibrosis” [34]. In our study, some sections were perceived as less applicable to some patients (e.g., the Spiritual Thoughts section may have been less helpful to non-religious participants, and the Life Support section may have been less helpful to currently healthy patients). There was nonetheless consensus around VMC’s value amongst the broader AYA population. The relatively lower ‘helpfulness to themselves’ ratings could be explained by the fact that the AYAs in our sample were survivors not currently undergoing active treatment. The phenomenon of optimism bias (or unrealistic optimism) [47]—the tendency of individuals to believe themselves less susceptible to negative events than others—may also have influenced these findings. Indeed, this bias may have interesting implications for introducing symptom control and quality-of-life topics to relatively healthy cancer patients earlier in their treatment trajectory.

Our study is the first to explore VMC’s sources of perceived, or anticipated, stress. Participants in our study considered the VMC Sections on Life Support and Being Remembered most stressful. Our thematic analysis further identified sources of perceived or anticipated stress arising from individual and interpersonal factors. Our findings, therefore, endorse the guide’s helpfulness whilst also acknowledging the potential for some stress (and distress) to arise in the course of these conversations. While distress is always important to manage and mitigate where possible, it is also a natural and understandable element of discussing end-of-life topics, particularly with young patients facing their mortality whilst on the cusp of the rest of their lives.

Distress in general (and anxiety in particular) can often be worse in anticipation of difficult events or conversations. The most recent data evaluating the American VMC guide indeed demonstrated that while ~50% of the AYA sample reported moderate/a lot of end-of-life-related anxiety at baseline, this reduced significantly to ~30% immediately after using the guide [27]. Additionally, in our study, the greatest anticipated stress was predicted by parents, followed by health professionals, with AYAs themselves reporting that they anticipated the lowest stress. We can speculate that this may reflect that parents’ and health professionals’ ratings of anticipated distress also incorporate their own meta-level distress around how they might help or support the AYA to manage this distress. These differences in anticipated distress could also reflect that AYAs have different perceptions of what they themselves feel they may be capable of emotionally coping with—potentially viewing themselves as more resilient in the face of these conversations than their parents or health professionals do. These hypotheses are worthy of further study. However, taken together with the US data, our findings suggest that guides such as VMC may help mitigate distress through the creation of certainty and a sense of personal control—in the form of preferences and plans—amidst the inherent uncertainty of advanced cancer and end-of-life decision making. By understanding the sources of stress outlined here, health professionals can adjust their practice to mitigate these stressors to the greatest extent possible.

### 4.1. Limitations

Several methodological limitations warrant consideration. Due to purposive sampling and opt-in-based recruitment approaches, we cannot calculate response rates and cannot rule out participant self-selection, resulting in our sample being especially passionate about and/or comfortable with end-of-life discussions. While our sample’s cultural composition was fairly well-matched to the Australian population (20% of our sample spoke a language other than English and 28% were born overseas vs. 21% and 32% of the Australian population, respectively) [48], we only recruited fluent English speakers and did not recruit any Indigenous Australians. Our sample was also more educated than the general population: 70% of our AYAs had completed tertiary education, compared with 42% and 51% of males and females aged 20–24 years in the Australian general population [49]. Finally, our AYA sample included post-treatment survivors. This enabled us to examine the perception of VMC’s acceptability in a lower-risk setting, mirroring a situation when end-of-life decisions are not imminently required. However, survivors were likely also physically healthier and less distressed than patients with incurable cancer approaching an end-of-life phase. Our specific sample of survivors also included a limited range of diagnoses.

### 4.2. Future Directions

Developing a culturally-tailored, Australian version of VMC is an important next step. Additionally, research needs to determine whether the VMC guide and approach is appropriate for sub-groups of Australians, such as culturally and linguistically diverse groups, and Indigenous AYAs, who experience poorer cancer outcomes compared to the general population of AYAs, [50] and who have unique needs to ensure culturally safe healthcare [51,52]. In particular, Australian health professionals report gaps in their comfort and experience communicating around spiritual and end-of-life topics with Indigenous Australian children receiving palliative and end-of-life care [53]. Further intersectional lenses could also be applied to research aimed at improving the acceptability of the Australian VMC for other minority groups with unique needs such as LGBTQI+ [54] and culturally and linguistically diverse communities [55]. Research led by, and in partnership with representatives and stakeholders from these communities is needed to better understand whether advance care planning communication guides like the Australian VMC tool would add value to the patient- and family-centric care delivered for all Australian patients and families—and if so, how these might need to be further tailored.

From a clinical perspective, excellent communication is a necessary—but not necessarily sufficient—ingredient to support high-quality, patient-centric palliative and end-of-life care. Data are therefore needed to determine whether age and culturally appropriate end-of-life communication guides lead to better end-of-life communication and more consistent delivery of goal-congruent end-of-life care for AYA patients and their families. Recent American data reported that AYA participants were more likely to have had end-of-life conversations after completing VMC [27,56]—which is suggestive of a flow-on effect to communicate with others outside of the clinical interaction in using the guide. The next stages of this research will explore the reception of VMC amongst AYAs in the later stages of their cancer treatment and explore methods to facilitate its clinical implementation.

Finally, research into the feasibility of integration of an Australian version of VMC into the Australian clinical context should be conducted. Further study into considerations such as the timing of introduction to advance care planning and documents like VMC would be helpful to ensure optimal integration. The variety of responses to the document in our study also suggest that an individualised approach should be taken, which is advocated for in other literature, along with an early approach to the introduction of these topics [57]. Further research on this and other implementation considerations would be a necessary next step.

### 4.3. Clinical Implications

By having identified sources of stress that most concern AYAs, parents, and health professionals, we can now address how these potential barriers can influence the use of VMC in Australia. For example, health professionals’ descriptions of what they perceive as the biggest source of stress also point to likely reasons why they may not want to introduce the guide. Table 5 outlines considerations for multidisciplinary health professionals introducing a future Australian VMC to address the potential for each of the stressors identified.

## 5. Conclusions

Australian AYAs with cancer, their parents, and health professionals view VMC as appropriate and helpful. Anticipated sources of stress in completing VMC—individual, developmental, and interactional stress—may be mitigated by health professionals taking care to introduce and support the guide’s facilitation in the context of a young person’s history and cancer care. Future research should explore how VMC can be adapted to best meet the needs of Australian AYAs from diverse cultural backgrounds and at different stages of treatment so that all AYAs may have the opportunity to discuss their end-of-life preferences in a well-supported way.

## Figures and Tables

**Table 1:** Sections of the original American Voicing My Choices (used with permission from Wiener et al., 2021 [27]) and the two additional proposed sections.

Number	Section Title	Abbreviated Title	Section Description/Purpose
ORIGINAL SECTIONS			
A	Introduction	Not applicable	Provides a brief overview of the guide while offering comfort and purpose to AYAs who may have mixed feelings about utilising a guide, informs AYAs that document completion is based on their thoughts and desires, and illustrates autonomy and respect for their ability to make decisions about their own care.
1	How I Want to be Comforted	Comfort	A place to describe how the AYA wants to be cared for and what will make them feel more comfortable (music, movies, food, atmosphere) in addition to preferences surrounding pain management.
2	How I Would Like to be Supported So I Don’t Feel Alone	Support	Allows the individual to describe who and when visitors are wanted, as well as designating how information on health status should be shared with others.
3	Who I Want to Make My Medical Care Decisions if I Cannot Make Them on My Own	Medical Decisions	Designating a durable power of attorney and expressing preferences for types of care decisions.
4	The Types of Life Support Treatment I Want, or Do Not Want	Life Support	A space for the individual to express their desire for/against life support treatments and to specify any terms on its usage, as well as to indicate where they would like to be at the end of their life.
5	What I Would Like My Family and Friends to Know About Me	Family and Friends	A space for the individual to express gratitude or regret towards family and friends.
6	My Spiritual Thoughts and Wishes	Spiritual Thoughts	A place to write down their thoughts on spiritual topics or sources of meaning, and to discuss their preferences.
7	How I Wish to be Remembered	Being Remembered	A space for the individual to designate burial and funeral preferences as well as provide information on organ donation and autopsy. This section also allows the individual to designate who important belongings should be distributed to and how they would like to be remembered on special occasions.
8	My Voice	My Voice	A space to write letters to loved ones.
B	Glossary	Not applicable	Provides definitions of terms used throughout the guide.
PROPOSED NEW SECTIONS			
9	Online Accounts	Online Accounts	Provides a place for AYAs to gather account information for use by friends and family, and indicate preferences around what they would prefer to happen with their social media accounts, in the event of the patient’s death.
10	VMC Storage	Storage	A page that allows AYAs to specify where they would like their copy of VMC to be kept and who should have access to it, per section as relevant.

Note: The introduction and Glossary were not evaluated in the present study.

**Table 2:** (a) Participant demographics across all groups. (b) Socio-demographic and cancer characteristics of AYA participants (*n* = 10). (c) Socio-demographic and cancer characteristics of parent participants (*n* = 5).

(a)					
		**AYAs** (***n* = 10**)	**Health Professionals** (***n* = 28**)	**Parents** (***n* = 5**)	**Total** (***n* = 43**)
**Age**	Mean (SD)	22.40 (3.2)	41.9 (9.6)	50.80 (3.9)	38.4 (12.3)
	Range	16–26 ^1^	25–61	46–56	16–61
Gender (*n* (%))	Male	4 (40)	5 (18)	1 (20)	10 (23)
	Female	6 (60)	23 (82)	4 (80)	33 (77)
Country of birth (*n* (%))	Australia	8 (80)	19 (68)	4 (80)	31 (72)
	United Kingdom	0	5 (18)	0	5 (12)
	New Zealand	1 (10)	1 (4)	0	2 (5)
	Other ^2^	1 (10)	3 (11)	1 (20)	5 (12)
(b)					
					**AYAs**
Highest education completed ^1^ *n* (%)		Year 10 or below			1 (10)
		Year 12			2 (20)
		TAFE certificate or diploma, college			1 (10)
		University degree			5 (50)
		Post-graduate degree			1 (10)
Current employment ^2^		Employed			8 (%)
		Unemployed			1 (%)
Language other than English spoken at home—*n* (%)		Yes			3 (30) ^3^
		No			7 (70)
Age at cancer diagnosis		Mean (SD)			17.10 (2.5)
		Range			15–23
Time since diagnosis		Mean (SD)			5.30 (2.7)
		Range			1–10
Cancer type ^4^ *n* (%)		Blood			5 (50)
		Solid			4 (40)
		Brain			1 (10)
Current cancer stage ^5^ *n* (%)		1			0
		2			1 (14)
		3			1 (14)
		3–4			1 (14)
		4			2 (29)
		N/A			2 (29)
Risk Level ^6^ *n* (%)		Low			1 (10)
		Intermediate			3 (33.3)
		High			1 (10)
		Unsure			3 (33.3)
Treatments received *n* (%)		Surgical removal of cancer			5 (50)
		Chemotherapy			8 (80)
		Radiotherapy			5 (50)
		Bone marrow/stem cell transplant			4 (40)
(c)					
					**Parents**
Highest education completed ^1^ *n* (%)		Year 10 or below			0
		Year 12			0
		TAFE certificate or diploma, college			3 (60)
		University degree			2 (40)
		Post-graduate degree			0
Current employment ^2^		Employed			1 (%)
		Unemployed			2 (%)
Language other than English spoken at home—*n* (%)		Yes			0
		No			5 (100)
Current age of child ^3^		Mean (SD)			19.33 (2.9)
		Range			16–21
Age of child when they died ^4^		Mean (SD)			17 (2.8)
		Range			15–19
Child age at cancer diagnosis		Mean (SD)			15.80 (3.3)
		Range			12–21
Child’s cancer type ^5^ *n* (%)		Blood			2 (40)
		Solid			1 (20)
		Brain			2 (40)
Child’s cancer stage ^6^ *n* (%)		1			2 (67)
		2			0
		3			0
		3–4			0
		4			1 (33)
		N/A			0
Child’s cancer risk level ^7^ *n* (%)		Low			1 (%)
		Intermediate			0 (%)
		High			3 (%)
		Unsure			0 (%)
Treatments received *n* (%)		Surgical removal of cancer			2 (40)
		Chemotherapy			5 (100)
		Radiotherapy			4 (80)
		Bone marrow/stem cell transplant			1 (20)

**Abbreviations and notes**: (a) **AYA**—Adolescents and young adults, **SD**—Standard deviation. ^1^ One participant became 26 years old between recruitment and interview date. ^2^ Other: included HCPs (*n* = 3)—Croatia, Spain, United States. AYAs (*n* = 1)—Fiji. Parents (*n* = 1)—South Africa. **TAFE**—Technical and Further Education. ^1^ Year 12 is the final year of high school in Australia. TAFEs are government-run vocational education and training institutions that provide tertiary (non-school) education. ^2^ One missing ^3^ One French, 1 Hindi, 1 Turkish ^4^ Blood: 3 Acute Myeloid Leukaemia, 2 Hodgkin’s lymphoma. Solid: 2 Bone sarcoma, 1 Epithelioid sarcoma, 1 Melanoma. ^5^ Three AYAs missing ^6^ Two AYAs missing. ^2^ One AYA and 2 parents missing. ^3^ Only for parents of AYA survivors (*n* = 3). ^4^ Only for bereaved parents (*n* = 2). ^5^ Blood included *n* = 1 Acute Myeloid Leukaemia, *n* = 1 Hodgkin’s lymphoma. Solid: *n* = 1 Bone sarcoma. ^6^ Only for parents of AYA survivors (*n* = 3). ^7^ One missing.

**Table 3:** Characteristics of health professional participants (*n* = 28).

Years of Experience	Mean (SD)	16.9 (7.8)
	Range	4–30
Healthcare profession—*n* (%)	Oncologist or Haematologist	8 (29)
	Nursing	11 (39)
	Clinical Psychologist	3 (11)
	Dietitian	1 (4)
	Social Worker	6 (21)
	Leisure Therapist	1 (4)
Specialty within oncology—*n* (%)	Paediatrics	15 (54)
	AYAs	4 (14)
	Adult	8 (29)
	Unspecified/missing	1 (4)
Estimated number of AYAs treated who have died from cancer—*n* (%)	<5	2 (7)
	5–10	3 (11)
	10–15	5 (18)
	15+	18 (64)

**Table 4:** Selected illustrative quotes reflecting sources of stressfulness in completing Voicing My CHOiCES.

Theme	Illustrative Quote
Intrapersonal: Individual Differences	
**Patient’s health**	“If it’s asked … (of) someone who’s got poorer prognosis then all of a sudden, to me, I would then be thinking this is the end, or this is when they’re really sick.” *(Comfort, Female, 38, Healthcare professional [HCP])*
**Prognostic awareness**	“It depends what experience they’ve been through. You know, if they’re a leukaemic who’s been through a huge amount of treatment, then they have a transplant and they’re failing, I think they can deal with it better than we think.” *(Life Support, Male, 60, HCP)*
**Higher distress, avoidant coping**	“In that beginning phase, you hold on to every little bit (of hope). You are so devastated by that ‘C’ diagnosis. …To think about it in advance and put it into her mind that she might need life support somewhere along the way, I wouldn’t even dream of it.” *(Life Support, Female, 46, Parent)*
**Desire to plan**	“Years ago, this little girl … she had lost her mum and she was dying, and she knew. She got an exercise book—she was 13—and she started writing stuff down. … This would have been perfectly right for her, but I think it would be very confronting for other people.” *(Being Remembered, Female, 39, HCP)*
**Spiritual beliefs**	“I looked around and I saw babies who were screaming. It was so horrible, I thought to myself there’s no way that there’s a God that would do this to people. Something like this (Spiritual section) while I was in my treatment … would have irritated me, because I would have been like, I don’t believe in this. I don’t think that it’s important at all. I just don’t want to be associated with it, and I just don’t want to do anything with it.” *(Spiritual Thoughts, Female, 24, Adolescent/young adult [AYA])*
**Intrapersonal: common AYA developmental factors**	
**Decision-making stress**	“These questions are good in that they are quite clear that (these are) moments when there is really not any hope … but I think any time that you’re thinking about life support there is that question of, “but what if I recover?” Like just being unable to predict.” *(Life Support, Female, 27, HCP)*
	“The ones who are like on that 17/18 (year-old) border I’ve just kind of seen them default to their parents because that’s what they’ve been doing so much of their lives.” *(Medical Decisions, Female, 27, HCP)*
	“This young man I worked with recently, he really struggled to find the words and put a lot of pressure on himself because he’s like, ‘mum’s going to read this over and over and over again. I don’t want to stuff it up’”. *(My Voice, Female, 34, HCP)*
**Complex concepts**	“Young people don’t plan their funeral and you’d never think you’d have to plan your funeral… As a young person, that’s not even in your mind, it’s not even a thought. So, to sit down and (ask), you know, “do you want an open casket, do you want a closed casket?”—I guess that, again, just drives home that something can go wrong.” *(Being Remembered, Male, 23, AYA)*
	“’If I can’t go to the bathroom’, … probably by their nature, young people haven’t had much exposure to that kind of stuff. Whereas for adults, if they have been caring for someone who’s deteriorating, they know what happens. … (It) might actually be quite distressing because they wouldn’t have pictured themselves in that space.” *(Comfort, Female, 35, HCP)*
	“Some of the questions—(about having) a limited autopsy, a standard autopsy, a research protocol autopsy—what would a young person know, feel, about an autopsy? And then actually there are four different autopsy choices there, as well as being an organ donor and donating my body to science.” *(Being remembered, Male, 48, HCP)*
	“If you’re told, ‘you’ve got this (disease), and we believe you’ve got 12 months to live’, or ‘Look, we believe that the treatment we’re going to give you (will) give you a really good chance of survival’—if you’ve been told that and then you’re given this (guide) … you might actually start to think it’s worse than what it is. ‘They’re not telling me the truth. I am going to die.’” *(S2, Female, 52, Parent)*
	“I wouldn’t want to think about the specifics. I mean, it doesn’t matter. I’m gone.” *(Online Presence Management, Female, 20, AYA, 20)*
**Interpersonal: Internal social perceptions: how AYAs think about their world**	
Social pressure	“Not necessarily intentional pressure, but I could see the young person having certain ideas and then feeling pressure that that (decision is) not what the parents or family might want.” *(Support, Female, 27, HCP)*
Impact on others	“The only thing I’m frightened by is in this section is probably the impact on the person that would be making those decisions for you (how they may cope).” *(Medical Decisions, Male, 26, AYA)*
Stress from the reflective process	“This young man … he wanted to apologise to his brother, and it was very stressful for him to find the right words. So, we (wrote letters) … together and bounced ideas around, but it brought up a lot of, I guess, regrets for him.” *(Friends and Family, Female, 34, HCP)*
	“If they’re trying to answer questions where they don’t have a support network … it’s like a sort of, a mirror back in your face letting you know that you don’t really have people to write down, so that can be upsetting.” *(Support, Female, 24, AYA)*
**Interpersonal: External social processes and how AYAs interact with their social connections**	
Conflicting opinions between patient and family	“Most (AYAs) can be really, really realistic about what their wishes are. It can be very stressful because maybe their parents are pushing for their treatment or pushing for any measures that can continue their life as long as possible. … I’ve had a patient where he was very up front to ask about what he wanted but then was continuing (treatment) for his parents.” *(Life Support, Female, 25, HCP)*
	“If someone comes from quite a religious or spiritual family, they may now be feeling that they want to voice something that may be different from their family (and) that could create some stress. … Their parents may say, “Oh no, we want the chaplain to come every day”, but the young person will actually (think), “No, I don’t want any of that”.” *(Spiritual thoughts, Female, 37, HCP)*
**How guide is introduced**	“If they were slowly eased into it with someone like a clinical psychologist (it would be better) … ‘Have your treatment and then if you want, we can do it now, or you can have a think about it and when you’re ready just tell me’. It’s [about] control again. ‘This is when *I* want to broach it; not when you want.’” *(Being Remembered, Male, 56, Parent)*

Experience of using the VMC guide: Benefit and burden.

**Table 5:** Clinical practice implications for mitigating sources of stressfulness in Voicing My CHOiCES.

Source of Stress	Clinical Implications for Health Professionals
Intrapersonal: Individual Differences	
Patient’s health	Lead AYAs (and their family members) into conversations about VMC early in the treatment trajectory by introducing broad concepts gently, and repeatedly checking the AYAs are comfortable proceeding with the conversation/getting their permission
Prognostic awareness	Check in with patients at the start of each conversation by asking (again) what the patient’s current understanding of their illness, treatment plan, and next steps is. This can include health professionals asking what patients recall of previous conversations, what they have continued to think about, and what else (worries, memories, prior experiences) may be playing on their minds with regard to their current health situation
Higher distress, avoidant coping	Regularly monitor and assess patients’ current physical- and symptom-related burden to enable early interventions and additional supportive care as needed. Conversations around palliative care topics including VMC could be framed as ways to understand their symptom/supportive care needs to enhance their current quality of life.
	Screen for and assess psychological distress among all AYA patients, including those with additional vulnerabilities due to the timepoint of their treatment trajectory (e.g., newly diagnosed, recently relapsed, other recent treatment setbacks). These times of additional distress can also be used as openings to raise The benefit of having conversations to understand AYAs’ perspectives on their care and quality of life, and also a time to set up an expectation of an honest dialogue with the health professional, including if and when difficult topics become relevant in the future
Spiritual beliefs	Open discussion about religion and what it means to the AYA before introducing the VMC spirituality page can provide them with context. Link with Indigenous health workers if appropriate.
**Intrapersonal: Common AYA developmental factors**	
Decision-making stress	Emphasise that the guide is meant to be a ‘living document’ that can change if and as the AYAs’ preferences and circumstances change. Suggest the AYA can start by filling it in with a pencil if this is an issue.
	Support AYAs to use blank pages at the back of VMC to jot down thoughts/ideas for topics they want further information, support, or discussion about.
	Assure AYA that they do not have to make immediate decisions about these things and that they can reach out to others to discuss and help with decision making.
Complex concepts	Explore with AYAs how much information they like to receive, and how they best process and understand information, as early as possible in the treatment trajectory (e.g., visual vs. verbal information delivery). Normalise the fact that these preferences for information and detail can and do change as their clinical situation and physical and emotional circumstances change, and repeatedly check in to clarify if their preferences for information and its discussion/delivery may have changed.
	Consider also using the ‘teachback’ or ask-tell-ask technique to check AYA’s current understanding, discuss new information, and clarify AYAs’ understandings of what has just been discussed. Engage multidisciplinary team in helping AYAs understand concepts within the guide, normalising their questions by using language such as “What doesn’t make sense?”, “What questions do you have?”, and “What could I/we explain better or differently?”. If appropriate in the context of the clinical relationship, consider asking the AYA to role-play with you and show how they might explain a particular concept/page to a close family/friend; e.g., “How would you explain what we just talked about to your sister/best friend?”
**Interpersonal: Internal social perceptions: how AYAs think about their world**	
Social pressure and impact on others	Normalise and provide psychosocial support around the fact that AYAs can feel very worried about making the ‘right’ decisions and/or causing stress, sadness, or conflict amongst family and friends. Discuss this proactively with AYAs in the context of psychosocial assessments as early as possible, and support AYAs with coping frameworks to balance considering (and prioritising) their own needs versus being concerned with others’ needs and expectations. Support AYAs to explore which key relationships in their lives they would like to involve in decision making and engage psychosocial team members in supporting AYAs with coping strategies to manage stress around any challenging relationships, and with communication strategies to broach trickier issues.
Stress from the reflective process	Screen and assess for psychological distress at key transition points of the cancer treatment trajectory (e.g., at the start of a transplant, upon receipt of new clinical information regarding the progression of the disease, or when discussing new treatment options). Normalise that different topics contained within the guide might lead the AYA to feel distressed, worried, or concerned, and highlight the benefits that can come through their discussion by enabling the AYA’s priorities to be supported and enabling the clarification of some certainty amidst the uncertainty. Normalise the range of psychological responses that can arise at different points on the cancer treatment trajectory and offer access to psychological support, regardless of whether AYAs have declined this in the past while recognising that distress levels and desire for help can fluctuate over time.
**Interpersonal: External social processes and how AYAs interact with their social connections**	
Conflicting opinions between patient and family	Meet with AYA and family, together and separately if and as appropriate, early in the treatment trajectory and throughout treatment. Name and normalise the fact that AYAs and family members frequently feel differently about aspects of cancer treatment and related decisions (can refer to research evidence regarding a discrepancy in AYA-parent perspectives if this is a clinically useful approach). Flag as early as possible the HCP’s role in terms of being open and honest with the patient and family, and that the HCP will not collude in ‘hiding’ or keeping secrets from AYAs. Use language such as ‘I wonder…’ to explore patient and family concerns about different options and future scenarios and language such as ‘I worry…’ to highlight potential risks to the patient’s wellbeing or care, for instance, certain topics not discussed or care options not considered.
How the guide is introduced	Identify which HCP on the multidisciplinary team has the strongest working relationship with the AYA to introduce the guide. Where relevant/appropriate, invite the AYA to take the lead on deciding which sections they may wish to complete with which HCPs.

**Abbreviations**: **AYA**—Adolescents and young adults, **HCP**—Healthcare professional, **VMC**—Voicing My CHOiCES^TM^.

## Data Availability

The data presented in this study are available on request from the corresponding author. The data are not publicly available to protect the privacy of participants.

## Supplementary Materials

The following supporting information can be downloaded at https://www.mdpi.com/article/10.3390/cancers15072129/s1, Table S1: Illustrative quotes regarding perceived sources of stressfulness in Voicing My CHOiCES; Table S2: AYAs’ perceptions of the benefits and burdens involved in completing Section 3 (Medical Decisions). Table S3: AYAs’ perceptions of the benefits and burdens regarding other VMC sections they chose to complete.

## Abbreviations

AYA	Adolescent and young adult
VMC	Voicing My CHOiCES
US	United States of America
