# Supplementary material for: Adapting the Voicing My CHOiCES Advance Care Planning Communication Guide for Australian Adolescents and Young Adults with Cancer: Appropriateness, Acceptability, and Considerations for Clinical Practice
Source: Cancers (Basel). 2023 Apr 3;15(7):2129. doi: 10.3390/cancers15072129 (PMC10093261; doi:10.3390/cancers15072129)
Supplement: Supplementary file 1 [file cancers-15-02129-s001.zip › cancers-2247908-supplementary.pdf]

cancers MDPI

Supplementary Materials

Adapting the Voicing My CHOiCES advance care planning communication guide for Australian adolescents and young adults with cancer: Appropriateness, acceptability, and considerations for clinical practice

Table S1. Illustrative quotes regarding perceived sources of stressfulness in Voicing My CHOiCES.

Category	Theme	Sub-theme	Illustrative Quote
Intra-personal: individual differences	Patient's current health	Too early	"If someone's quite pre that stage whether or not that's something they've thought about… it could actually seem more distressing than what they'd anticipated." *(Comfort, Female, 37, HCP)*
		Too late	"If it's asked (to) … someone who's got poorer prognosis then all of a sudden, to me, I would then be thinking this is the end, or this is when they're really sick." *(Comfort, Female, 38, HCP)*
	Patient's prognostic awareness	Prognostic awareness	"I wouldn't be doing a page like this with someone who didn't know their treatment was not curative, … someone who isn't comfortable to talk about it." *(Life Support, Female, 39, HCP)*
		Previous experiences	"It depends what experience they've been through. You know, if they're a leukaemic who's been through a huge amount of treatment, (and) then they have a transplant and they're failing, I think they can deal with it better than we think." *(Life Support, Male, 60, HCP)*
		Previous conversations	"It really depends on what conversation has happened prior… Some of the young people I've worked with (have) had the conversation, they know what's going on, they really want to know about the dying process, so this (would have been) really important to them." *(Comfort, Female, 34, HCP)*
	Higher distress & avoidant coping	Treatment burden and intensity	"When I was going through treatment, it never once crossed my mind - about a funeral or what's going to happen to me once I pass away. … If you're going through treatment (those thoughts) can be quite overwhelming and just quite negative. … I'd rather focus on positive … It sort of doesn't give you any hope when you're confronted with questions like that." *(Being Remembered, Female, 24, AYA)*
		Recency of diagnosis	"In that beginning phase, you hold on to every little bit (of hope). You are so devastated by that 'C' diagnosis. …To think about it in advance and put it into her mind that she might need life support somewhere along the way, I wouldn't even dream of it." *(Life Support, Female, 46, Parent)*
	Communication style	Openness to discussing feelings	"(These questions are) really talking about their feelings and some (people), whether they were sick or not were never going to be comfortable doing that. Things like 'things that give me strength' and 'things that give me joy', it's almost very grown-up language." *(Friends & Family, Female, 38, HCP)*
	Desire to plan	Lifestyle habits	"Depending on the person (some might) find it a relief and a good thing … to have a sense that there are some plans in place." *(Being Remembered, Female, 55, HCP)*
		Stage of acceptance	"Years ago, this little girl … she had lost her mum and she was dying, and she knew. She got an exercise book - she was 13 - and she started writing stuff down. … This would have been perfectly right for her, but I think it would be very confronting for other people." *(Being Remembered, Female, 39, HCP)*
	Spiritual beliefs	Irritation and offence towards religion	"I looked around and I saw babies who were screaming. It was so horrible, I thought to myself there's no way that there's a God that would do this to people. Something like this (section) while I was in my treatment … would have irritated me, because I would have been like, I don't believe in this. I don't think that it's important at all. I just don't want to be associated with it, and I just don't want to do anything with it." *(Spiritual Thoughts, Female, 24, AYA)*
		Indigenous background	"I recently learnt that people from (some) Indigenous backgrounds find questions about the afterlife associated with cancer, a bit (irrelevant and) distressing … because when they get affected by cancer, they don't necessarily treat it. Cancer just means death." *(Spiritual Thoughts, Female, 23, AYA)*
Intra-personal: common AYA developmental factors	Decision-making stress	Not wanting control	"The ones who are like on that 17/18 (year-old) border I've just kind of seen them default to their parents because that's what they've been doing so much of their lives." *(Medical Decisions, Female, 27, HCP)*
		Big decisions	"You're brought up - you go to the doctor, and you do what the doctor tells you. So, to be able to stop previously started treatment, or to 'hire or fire' a healthcare worker - that's stuff that's like, wow - hold on - that's a bit…" *(Medical Decisions, Female, 52, Parent)*
		Finality of completing VMC	"This young man I worked with most recently, he really struggled to find the words and put a lot of pressure on himself because he's like, "mum's going to read this over and over and over again. I don't want to stuff it up"." *(My Voice, Female, 34, HCP)*
			"I wouldn't want to write in it until I knew exactly what I was writing. I'd want a draft… just because it's a part of the booklet it would stress me out more because I'm like, this is the final thing. I'd rather write on loose paper then when I'm happy with it (copy it over)." *(My Voice, Female, 20, AYA)*
		Uncertainty of the future	"These questions are good in that they are quite clear that (these are) moments when there is really not any hope, … but I think any time that you're thinking about life support there is that question of, "but what if I recover?". Like just being unable to predict." *(Life Support, Female, 27, HCP)*
		VMC's formatting and framing of questions	"Why do you have to have very specific answers (for) 'The people I would like to come visit me'? … You'd struggle to get that out of most 17/18-year-olds. They'll just say, "Yeah, I mean I'm still connected to the church through my parents, and I wouldn't mind it if people came". They wouldn't say, "Oh, yeah, I want him, and I want him". It's very unusual in my experience." *(Spiritual Thoughts, Male, 61, HCP)*

			“The wording around ‘Other thoughts I have about treating my pain or helping me make…’ - maybe being a bit more directive about, ‘If I’m in pain, I would like…’, just because, particularly for young adolescents, they might struggle with “Oh, what would be another thought that I would be having”.” *(Comfort, Female, 35, HCP)*
			“I feel like I wouldn’t know how to answer some of them, like the ‘Things that are important to know about me’. That one stresses me out. … I just felt like it's a bit vague and a bit of pressure to have something - like I want to say something, but I'm like I don’t know.” (Family & Friends, Female, 20, AYA)
		Too many decisions to make	“Young people shouldn't have to think about planning a funeral and it's just a lot of things to think about.” *(Being Remembered, Female, 23, AYA)*
			“I wouldn't want to think about the specifics. I mean, it doesn't matter. I'm gone.” *(Online Presence Management, Female, 20, AYA)*
			“It might open them up to just how many different things they have to think about maybe in the future.” *(Medical Decisions, Male, 25, AYA)*
		Areas they have not thought about before	“If they've been in hospital for a long time, I feel like sometimes the default answer for a lot of teens is “I'm bored and I'm not happy and there's nothing I can do to change it”. Whereas these questions are asking what's going to make you comforted and perhaps it hasn't been something that they've specifically identified before. So, I feel like that might be stressful if they're so unwell that we're bringing this up and they haven't identified this previously.” *(Comfort, Female, 27, HCP)*
	Confronting or complex concepts	End of life, death, afterlife	“Probably the last question ‘when the end of my life is near’, … I think no matter how you frame that it's going to create some stress for the young person.” *(Support, Female, 37, HCP)*
			“Young people don't plan their funeral and you'd never think you'd have to plan your funeral… As a young person, that's not even in your mind, it's not even a thought. So, to sit down and, you know, “do you want an open casket, do you want a closed casket?” - I guess that, again, just drives home that something can go wrong.” *(Being Remembered, Male, 23, AYA)*
			“I think it’s normal to think about these things, but when I was going through treatment, it never once crossed my mind - about a funeral or what’s going to happen to me sort of once I pass away. … I just think that if you’re going through treatment (those thoughts) can be quite overwhelming and just quite negative. … I’d rather focus on positive … (because) it sort of doesn’t give you any hope when you’re confronted with questions like that.” (Being Remembered. Female. 24, AYA)
		Loss of function or control	“’If I can’t go to the bathroom’, … probably by their nature, young people haven’t had much exposure to that kind of stuff. Whereas for adults, if they have been caring for someone who’s deteriorating, they know what happens. … (It) might actually be quite distressing because they wouldn’t have pictured themselves in that space.” *(Comfort, Female, 35, HCP)*
			“The thought of not being able to make decisions for yourself and having all of that control… being taken away from you.” *(Life Support, Female, 16, AYA)*
			“It says ‘I want treatment to help me if I look sad and irritable, nauseated, confused,’ … A lot of (AYAs) don’t want to communicate at certain times, but at the same time they might not want people to do things without them vocalizing it. … (If they’re) looking nauseated and someone starts giving them medicine, they’re like “hang on, what are you doing?”.” *(Comfort, Male, 35, HCP)*
		Terminology	“For example, … under ‘tracheotomy’ in the glossary, all it says is ‘a surgical operation that creates an opening in the windpipe with a tube inserted to provide a passage for air in order to help someone breathe’. That’s a very clinical description of something that impacts significantly on somebody’s life, and you can’t ask this (glossary/booklet) a question.” *(Life Support, Male, 35, HCP)*
			“Some of the questions - a limited autopsy, a standard autopsy, a research protocol autopsy - what would a young person know, feel about an autopsy? And then actually there are four different autopsy choices there, as well as being an organ donor and donating my body to science.” *(Being Remembered, Male, 48, HCP)*
		Confusion about purpose of guide	“If you're told, “you’ve got this (disease), and we believe you’ve got 12 months to live”, or … “Look, we believe that the treatment we're going to give you is going to give you a really good chance of survival” - if you’ve been told that and then you're given this… you might actually then start to think it's worse than what it is. They're not telling me the truth. I am going to die.” *(Support, Female, 52, Parent)*
			“Aren't you giving the person the impression that well, we're just tidying things up around your death? … I can tell you this is about (legally supporting) deliverers of clinical care. It's not about supporting the patient. He's dying; he doesn't really care what happens. As a doctor caring for him, I absolutely have to know whether they can sue me if I don't put him on a ventilator.” *(Life Support, Male, 61, HCP)*
Interpersonal: Internal social perceptions - how AYAs think about their world	Fear of offending others	Perceived pressure	“Not necessarily intentional pressure, but I could see the young person having certain ideas and then feeling pressure that that (decision is) not what the parents or family might want.” *(Support, Female, 27, HCP)*
	Impact on family and friends	Causing sadness	“It also has the scope to make you feel concerned about how your loved ones are going to be feeling after your death, and that can be really upsetting.” *(Being Remembered, Male, 49, HCP)*
			“I think it's always hard for a young person or for anyone to say to a family member or a friend, not today - especially when they've made an effort to come.” (Support, Female, 47, HCP)
			“It is stressful because … they're thinking about … not just (what) they want … because it says about if people are upset or crying and it's taking it outside of themselves.” (Support, Female, 42, HCP)
		Causing stress	The only thing I’m frightened by is in this section is probably the impact on the person that would be making those decisions for you (how they may cope).” *(Medical Decisions, Male, 26, AYA)*
			“You might be saying I really prefer these people and not these other people. Maybe that feels a bit as though you're picking and choosing.” (Support, Female, 49, HCP)
	Stress from the reflective process	VMC process too emotive and reflective	“This young man… he wanted to apologise to his brother, and it was very stressful for him to find the right words. So, we (wrote letters) … together and bounced ideas around, but it brought up a lot of, I guess, regrets for him.” *(Friends & Family, Female, 34, HCP)*
		Negative memories	“It's interesting that the first … question about needing to be forgiven is about themselves first, which just feels a little bit insensitive and a bit hard and confronting. This poor young persons in this situation and (has) to think about where (they’ve) failed or let someone down.” *(Friends & Family, Female, 37, HCP)*
		Lack of social support	“If they’re trying to answer questions where they don’t have a support network … it’s like a sort of, a mirror back in your face letting you know that you don’t really have people to write down, so that can be upsetting.” *(Support, Female, 24, AYA)*

	Internal social perceptions	Family and friends	“I struggled for a long time, how I thought my family and friends perceive me, how they reacted. It just makes me nervous when I think about them.” *(Family & Friends, Female, 20, AYA)*
	Concern about lack of privacy	Others invading their privacy	“They'd be freaking out at the same time that people are invading in their privacy … they completely lose control in these situations. It's a way to take some control back but at the same time we're ripping them apart I think.” *(Online Presence Management, Female, 41, HCP)*
Interpersonal: External social processes - how AYAs interact with their social connections	Conflicting opinions between the patient and their family	Families not accepting the possibility of death: seeking permission to begin palliation	“Sometimes because the parents or the siblings can't get (to the point of accepting the possibility of death) the child feels that they're not - they don't have permission to get there, and I think that permission to die … and permission to express your feelings before you die is very important.” *(Friends & Family, Female, 32, HCP)*
			“It can be quite difficult (for) the families to take in. … As soon as they hear something (distressing) they shut down and don’t want to continue on with the conversation. Again, it's really depending - you may have a family that will just embrace the palliation process and be open to this.” *(Life Support, Female, 34, HCP)*
			“Most (AYAs) can be really, really realistic about what their wishes are. It can be very stressful because maybe their parents are pushing for their treatment or pushing for any measures that can continue their life as long as possible. … I've had a patient where he was very up front to ask about what he wanted but then was continuing for his parents.” *(Life Support, Female, 25, HCP)*
		Religious views	“If someone comes from quite a religious or spiritual family, they may now be feeling that they want to voice something that may be different from their family (and) that could create some stress. … Their parents may say, “Oh no, we want the chaplain to come every day”, but the young person will actually (think), “No, I don't want any of that”.” *(Spiritual Thoughts, Female, 37, HCP)*
		Medical options	“I think the autopsy stuff could be pretty challenging, because I think there's possibly a limited amount of understanding. … Some of it may be against (the wishes of the) mum and dad or carer. I think that may cause a little bit of either anxiety or they won't fill it out appropriately.” *(Being Remembered, Female, 41, HCP)*
	Social complexities		“I'm sure there's going to be young people who don't want their default, their parent or guardian, to be making those decisions.” *(Medical Decisions, Female, 27, HCP)*
	Administration of VMC	With human support	“It would … (be) dependent on you setting the scene and having that person walking you through it, … someone else who can give it with (an) explanation rather than just throw it as a questionnaire.” *(Comfort, Female, 49, HCP)*
		Under the AYA’s control	“If they were slowly eased into it with someone like a clinical psychologist (it would be better) … ““Have your treatment and then if you want, we can do it now, or you can have a think about it and when you're ready just tell me”.” … “It's control again. This is when I want to broach it; not when you want.” *(Being Remembered, Male, 56, Parent)*

Abbreviations: **AYA** – Adolescents and young adults, **HCP** – Healthcare professional, **VMC** – Voicing My CHOiCES^{TM}.

Table S2. AYAs′ perceptions of the benefits and burdens involved in completing Section 3 (Medical Decisions).

**Benefit**		**Completed by (#AYAs)**	**Burden**		**Completed by (#AYAs)**
**Mean (SD)**	**Range**		**Mean (SD)**	**Range**	
2.89 (1.27)	0-4	9	1.13 (1.13)	0-3	8

**Reason**	**Selected by (#AYAs)**	**Reason**	**Selected by (#AYAs)**
Helped me think about/decide what I want	6	Thinking about the topics discussed made me anxious	3
It was helpful/relieving to voice my thoughts	5	Questions were confusing	2
It made me feel comfortable to share my thoughts	4	Questions were difficult to answer	1
Other[1]	4	Too many questions/section too long	1
Provided opportunity to discuss their wishes with others	3	The questions were upsetting	0
It addresses issues important to me	3	Other	0

Abbreviations: **AYA** – Adolescents and young adults, **SD** – Standard deviation. AYA participants assessed benefit and burden on a scale of 0-4 (0=not at all burdensome/beneficial to me, 1=a little bit, 2=somewhat, 3=quite a bit, 4=very much).

[1] Other reasons:

- Was helpful to think about topics they have not considered/thought about before. Previously parents took care of everything (given by 2 AYAs)
- Sense of control (1 AYA)
- Beneficial for the AYA’s parents (1 AYA)

Table S3. AYAs′ perceptions of the benefits and burdens regarding other VMC sections they chose to complete.

**Section**	**Benefit**		**Burden**		**Completed by (#AYAs)**
	**Mean (SD)**	**Range**	**Mean (SD)**	**Range**	
**1 – Comfort**	3.5 (0.58)	3-4	0 (0)	0-0	4
**2 – Support**	3	N/A	0	N/A	1
**4 – Life Support**	4 (0)	4-4	1.5 (1.92)	0-4	4
**5 – Know**	3.5 (0.71)	3-4	0 (0)	0	2
**6 – Spirit**	0	N/A	1	N/A	1
**7 – Remembrance**	2.75 (1.26)	1-4	2.0 (1.16)	1-3	4
**8 – My Voice**	N/A	N/A	N/A	N/A	0

Abbreviations: **AYA** – Adolescents and young adults, **SD** – Standard deviation, **VMC** – Voicing My CHOiCES^{TM}. AYAs completed two extra pages of VMC of their choice (in addition to Section 3 – Medical Decisions, displayed in Table 6a). Participants assessed benefit and burden on a scale of 0-4 (0=not at all burdensome/beneficial to me, 1=a little bit, 2=somewhat, 3=quite a bit, 4=very much).
